# Microbial community modulates growth of symbiotic fungus required for stingless bee metamorphosis

**DOI:** 10.1371/journal.pone.0219696

**Published:** 2019-07-25

**Authors:** Camila Raquel Paludo, Gleb Pishchany, Andres Andrade-Dominguez, Eduardo Afonso Silva-Junior, Cristiano Menezes, Fabio Santos Nascimento, Cameron R. Currie, Roberto Kolter, Jon Clardy, Mônica Tallarico Pupo

**Affiliations:** 1 School of Pharmaceutical Sciences of Ribeirão Preto, University of São Paulo, Ribeirão Preto, SP, Brazil; 2 Department of Microbiology, Harvard Medical School, Boston, MA, United States of America; 3 Brazilian Agricultural Research Corporation, Embrapa Meio Ambiente, Jaguariúna, SP, Brazil; 4 Department of Biology, FFCLRP, University of São Paulo, Ribeirão Preto, SP, Brazil; 5 Department of Bacteriology, University of Wisconsin, Madison, WI, United States of America; 6 Department of Biological Chemistry and Molecular Pharmacology, Harvard Medical School, Boston, MA, United States of America; Universidade Federal de Uberlândia, BRAZIL

## Abstract

The Brazilian stingless bee *Scaptotrigona depilis* requires the brood cells-associated fungus *Zygosaccharomyces* sp. as steroid source for metamorphosis. Besides the presence of *Zygosaccharomyces* sp., other fungi inhabit *S*. *depilis* brood cells, but their biological functions are unknown. Here we show that *Candida* sp. and *Monascus ruber*, isolated from cerumen of *S*. *depilis* brood provisions, interact with *Zygosaccharomyces* sp. and modulate its growth. *Candida* sp. produces volatile organic compounds (VOCs) that stimulate *Zygosacchromyces* sp. development. *Monascus ruber* inhibits *Zygosacchromyces* sp. growth by producing lovastatin, which blocks steroid biosynthesis. We also observed that in co-cultures *M*. *ruber* inhibits *Candida* sp. through the production of monascin. The modulation of *Zygosaccharomyces* sp. growth by brood cell-associated fungi suggests their involvement in *S*. *depilis* larval development. This tripartite fungal community opens new perspectives in the research of microbial interactions with bees.

## Introduction

The Brazilian stingless bee *Scaptotrigona depilis* (Moure, 1942) (Hymenoptera, Apidae, Meliponini) requires the fungus *Zygosaccharomyces* sp. to develop. This fungus, which start to grow inside brood cells after eggs hatch, is ingested by the larvae and is an essential source of ergosterol for ecdysteroid production [1]. Insects are unable to produce steroid *de novo* and all steroids they need are obtained from the food source, including those requested to produce ecdysteroids for pupation [2].

The association of *S*. *depilis* and *Zygosaccharomyces* sp. is an unprecedented case of bee-fungus symbiosis in Apidae, and offers a new perspective regarding pollinator-microbiota interaction. Considering the alarming disappearance of pollinators worldwide [3–5], studies involving bee-associated microorganisms could help the development of strategies to protect the bees. For example, by understanding the roles of symbiotic microbiota for these pollinators we can evaluate the impacts of pesticides directly against these microbes. The toxicity of pesticides for the microbial communities can disturb the colony fitness causing bee mortality in an indirect way [6].

Studies involving the microbiota associated with the honeybee *Apis mellifera* have demonstrated the importance of microorganisms to the bee health and susceptibility to parasites [6,7]. Stingless bees are the largest group of eusocial bees on the planet, with some 60 genera already described [8], however the knowledge about associated microbiota is still limited. Most of the studies described the isolation of microbial strains [9–15], and some protective roles of associated bacteria are also suggested [9–11].

The lack of information about the roles of the microbial communities isolated from stingless bees’ colonies prompted us to further study the microbiota associated with *S*. *depilis* brood cells. Besides *Zygosaccharomyces* sp., other fungi are found in the brood provisions of this stingless bee. *Monascus* spp. have been isolated from *S*. *depilis* and *Melipona scutellaris* colonies in different states in Brazil [1,12,13]. *Candida* spp. were also isolated from different stingless bees’ colonies [14,15], and their ecological functions are already unknown. The recurrently isolation of these fungi from stingless bees’ colonies indicates a possible symbiotic association.

By studying species that are repeatedly isolated from the same environment, we can select relevant members from a microbial community and characterize the ecological interactions among these microorganisms. In nature, microbes frequently engage in remarkable competitive and cooperative interspecies interactions [16]. These microorganisms can produce a range of small molecules that mediate microbe-host and microbe-microbe interactions. Insect-associated microorganisms can contribute with their host nutrition, providing essential amino acids, B vitamins and sterols [17]. Microbes can also inhibit specialized pathogens via antimicrobial compounds production, such as actinomycetes symbionts of fungus-growing ants, termites and beetles [18].

Our initial hypothesis was that *Zygosaccharomyces* sp., *Monascus ruber* and *Candida* sp., repeatedly isolated from brood cells of *S*. *depilis*, should interact among themselves via small molecules production. By simplifying this microbial community in laboratory, we addressed key roles that these fungi can play inside their host colonies. Through different multispecies culturing experiments, we characterized a complex tripartite fungal interaction that may be involved in the survival of the stingless bee *S*. *depilis*.

## Results and discussion

*Monascus ruber*, *Candida* sp. and *Zygosaccharomyces* sp. have been isolated from different brood cells of *S*. *depilis* colonies. To understand how they interact we selected the strains *Zygosaccharomyces* sp. SDBC30G1, *M*. *ruber* SDCP1 and *Candida* sp. SDCP2 to perform co-cultures and monocultures. *Zygosaccharomyces* sp. SDBC30G1 was isolated by collecting fungal cells directly from brood cells (S1 Fig). *M*. *ruber* SDCP1 and *Candida* sp. SDCP2 were isolated from *S*. *depilis* cerumen found in brood cells (S1 Fig).

**Fig 1:**
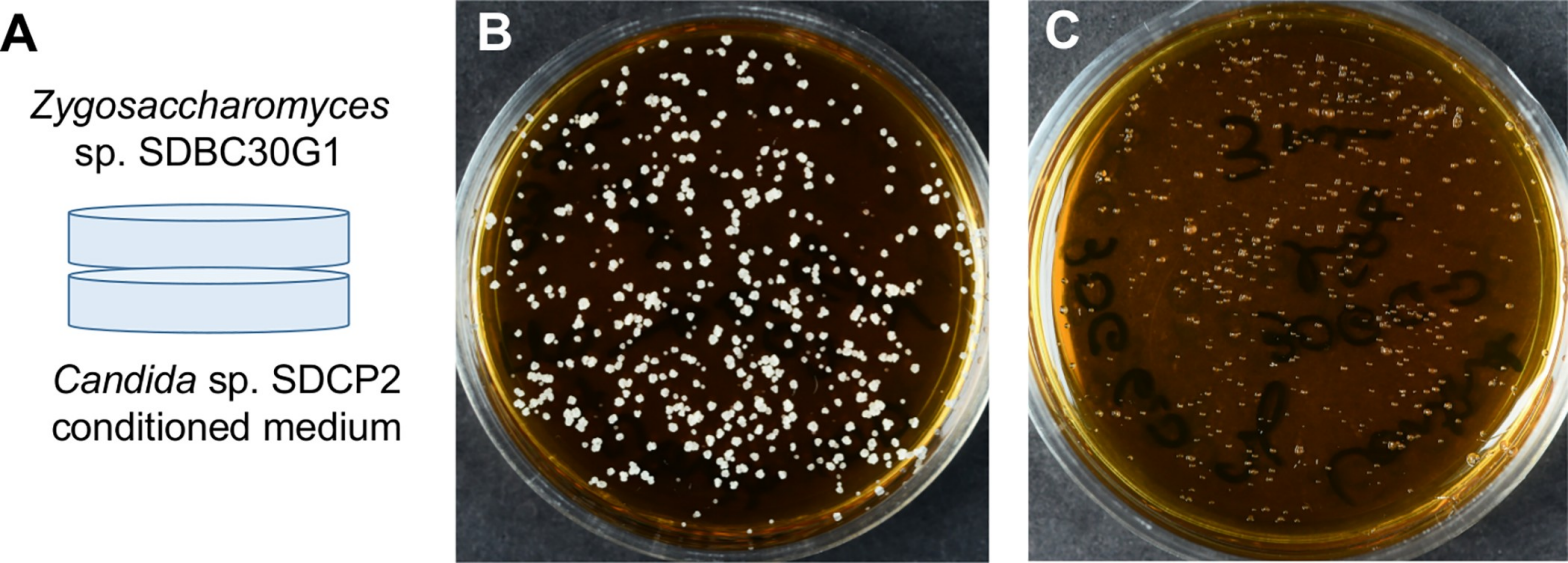
Interactions between *Zygosaccharomyces* sp. SDBC30G1 and *Candida* sp. SDCP2 VOCs. (A) Diagram illustrating the procedure where the cell-free supernatant of *Candida* sp. SDCP2 was transferred to a Petri dish and, on top of it, a fresh inoculated *Zygosaccharomyces* sp. SDBC30G1 was placed. (B) Culture of *Zygosaccharomyces* sp. SDBC30G1 after the contact with VOCs from *Candida* sp. SDCP2. (C) Culture of *Zygosaccharomyces* sp. SDBC30G1 after exposure to VOCs from unconditioned culture medium.

In co-culture using Petri dishes, we observed that *Candida* sp. SDCP2 stimulates the growth of *Zygosaccharomyces* sp. (S2A and S2B Fig). When we spotted the cell-free supernatant from *Candida* sp. SDCP2 onto the agar plates containing *Zygosaccharomyces* sp. SDBC30G1 inoculum, we also observed growth stimulation. In these experiments, the growth stimulus provided by the supernatant was not restricted to the area where it was spotted, but distributed throughout the dish, suggesting that volatile organic compounds (VOCs) were responsible for growth stimulation (S2C Fig). To evaluate this observation, we performed an experiment where the media conditioned by *Candida* sp. SDCP2 did not have physical contact with *Zygosaccharomyces* sp. SDBC30G1 culture. In this experiment, just VOCs could be responsible for phenotype changes on *Zygosaccharomyces* sp. SDBC30G1. As negative control, we used unconditioned broth. Using this method, we determined that VOCs from *Candida* sp. SDCP2 promoted the growth of *Zygosaccharomyces* sp. SDBC30G1. The number of colony forming units (CFU) did not change (1.7x10^4^ CFU/mL) (Figs 1 and S2D). However, the colonies grew faster, were bigger and presented floating phenotype in the presence of the stimulatory VOCs from *Candida* sp. SDCP2 (Figs 1 and S2D). Analyses by gas chromatography-mass spectrometry (GC-MS) of the active supernatant showed that ethanol was the most abundant VOC, followed by isoamyl alcohol (S3A–S3D Fig). Analyses of VOCs directly extracted from *S*. *depilis* brood cells revealed ethanol as the major compound as well (S4A–S4C and S4E Fig). Isoamyl alcohol was also detected in brood cells (S4A, S4B and S4D Fig). These results demonstrate that *Candida* sp. SDCP2 produces ethanol and isoamyl alcohol that stimulate the growth of *Zygosaccharomyces* sp. *in vitro*. Both volatiles are present in brood cells suggesting that they stimulate growth of *Zygosaccharomyces* sp. in the natural environment.

Ethanol has been reported to stimulate microbial growth in other studies [19,20]. Cell-free supernatants of *Aureobasidium pullulans* stimulated the growth of *Armillaria mellea*, and ethanol was the unique VOC detected by GC-MS analysis [19]. In another report, the ectomycorrhizal fungus *Boletus virigatus* produced ethanol that stimulated the growth of the root pathogenic fungi *Phytophthora cinnamomi* and *Fomes annosus* [20]. Other alcohols can modulate fungal growth and morphology. For example, *Saccharomyces cerevisiae* secrets aromatic alcohols involved in quorum-sensing signaling that promote its filamentous growth [21].

Additionally, we observed that *Candida* sp. SDCP2 decreases the pH of the medium while growing. When cultured in Potato Dextrose Broth (PDB), this fungus decreased the pH from 5.0 to pH 3.0, after 3 days of culturing. *Zygosaccharomyces* sp. SDBC30G1 grows better in acidic pH (S2A and S2B Fig), which is consistent with the pH of *S*. *depilis* larval food (pH 3.16 ± 0.039). This result indicates that *Candida* sp. SDCP2 could also facilitate *Zygosaccharomyces* sp. growth by decreasing the pH of the media.

**Fig 2:**
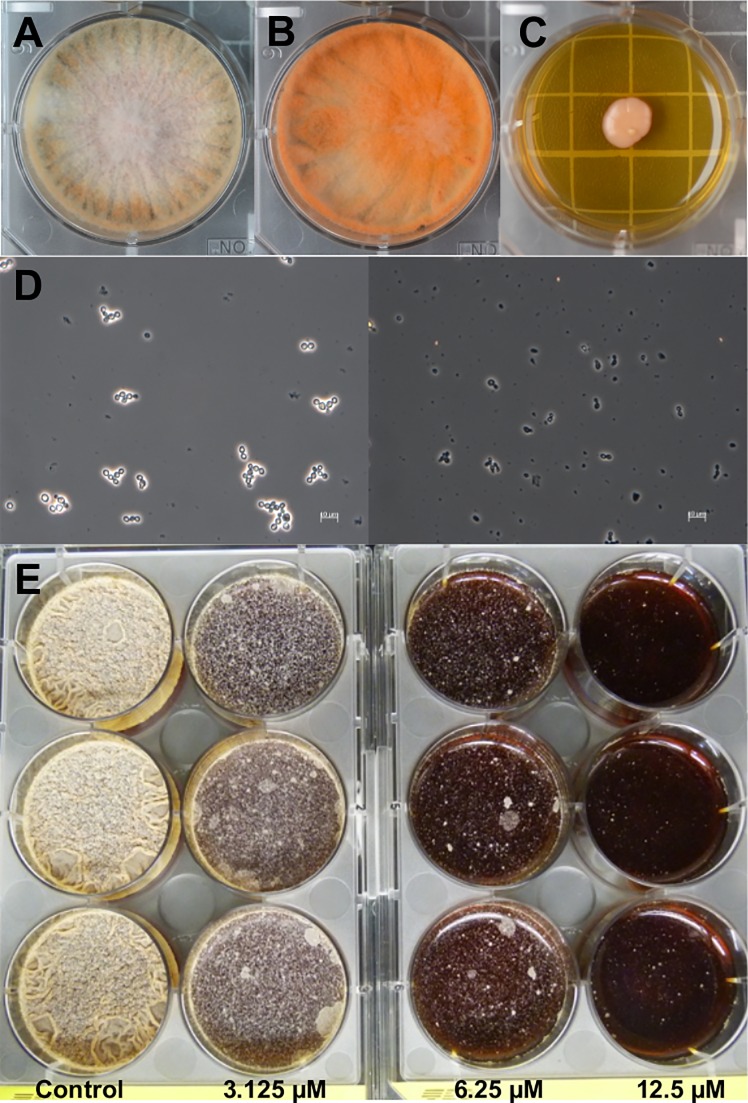
The effects of *Monascus ruber* SDCP1 secondary metabolites on *Candida* sp. SDCP2 and *Zygosaccharomyces* sp. SDBC30G1 growth. (A) *Monascus ruber* SDCP1 monoculture. (B) *Monascus ruber* SDCP1 cultured with *Candida* sp. SDPC2. (C) *Candida* sp. SDPC2 monoculture. (D) *Candida* sp. SDPC2 cells in monoculture (left) and co-culture with *Monascus ruber* SDCP1 (right) in liquid medium (scale 10 μm). (E) Lovastatin (3.125 μM, 6.25 μM and 12.5 μM) inhibitory effects on *Zygosaccharomyces* sp. SDBC30G1 growth in liquid medium.

To investigate the interactions between *Candida* sp. SDCP2 and *M*. *ruber* SDCP1, we also performed co-cultures. We observed that *Candida* sp. stimulated *M*. *ruber* SDCP1 pigment production (S1 Video, Figs 2A–2C and S5A), which did not occur during co-culture of *M*. *ruber* SDCP1 and *Zygosaccharomyces* sp. SPBC30G1 (S5A Fig). LC-HRESIMS analyses showed that these pigments were monascin and monascinol. The production of monascinol increased 3-fold compared to the amount produced in a monoculture of *M*. *ruber* SDCP1, while monascin production increased approximately 57 times in the co-culture (S5B–S5E Fig).

Monascinol, also known as monascuspiloin, and monascin are found in *Monascus* spp. fermented-rice, consumed in several countries of Asia [22,23]. These two related compounds have several biological activities, including anticancer [23] and anti-inflammatory [24]. We tested the antifungal activities of these polyketides and we found that monascinol did not inhibit *Candida* sp. SDCP2 or *C*. *albicans* ATCC MYA-2876 at concentrations below 400 μg/mL. However, monascin showed selective minimal inhibitory concentration (MIC) of 50 μg/mL and minimal fungicidal concentration (MFC) of 100 μg/mL against *Candida* sp. SDCP2.

*Monascus ruber* SDCP1 starts to produce monascin and monascinol on the second day in co-culture, and on the third day of co-culturing, the cells of *Candida* sp. SDCP2 are no longer viable, as confirmed by microscopy (Fig 2D) and culturing. These data indicate that *M*. *ruber* SDCP1 modulates the growth of *Candida* sp. SDCP2 by producing monascin, controlling the super population of this microorganism. However, as the fungicidal concentration of monascin is high (100 μg/mL), the coexistence of *Candida* sp. and *M*. *ruber* is possible inside *S*. *depilis* colonies.

*Monascus* spp. are also known to produce lovastatin (monacolin K), which reduces endogenous cholesterol biosynthesis. This natural product inhibits 3-hydroxy-3-methylglutaryl-coenzyme A reductase (HMG-CoA reductase), which catalyzes the production of mevalonate, a key intermediate for sterol biosynthesis [25]. Insects lack the enzymes to synthesize sterols [2,26]. They must consume all sterols they need from the diet and *Zygosaccharomyces* sp. is an essential source of sterols for *S*. *depilis* larvae [1]. We verified that *M*. *ruber* SDCP1 produces lovastatin when cultured in monoculture using a 30% glucose medium (S6A–S6C Fig). In liquid cultures, lovastatin decreased the growth of *Zygosaccharomyces* sp. SDBC30G1 (Fig 2E). These results indicate a modulatory function of *M*. *ruber* SDCP1 in the symbiotic system. *Monascus* spp. have been found in other Brazilian stingless bee colonies [13], and similar modulatory roles could occur.

**Fig 3:**
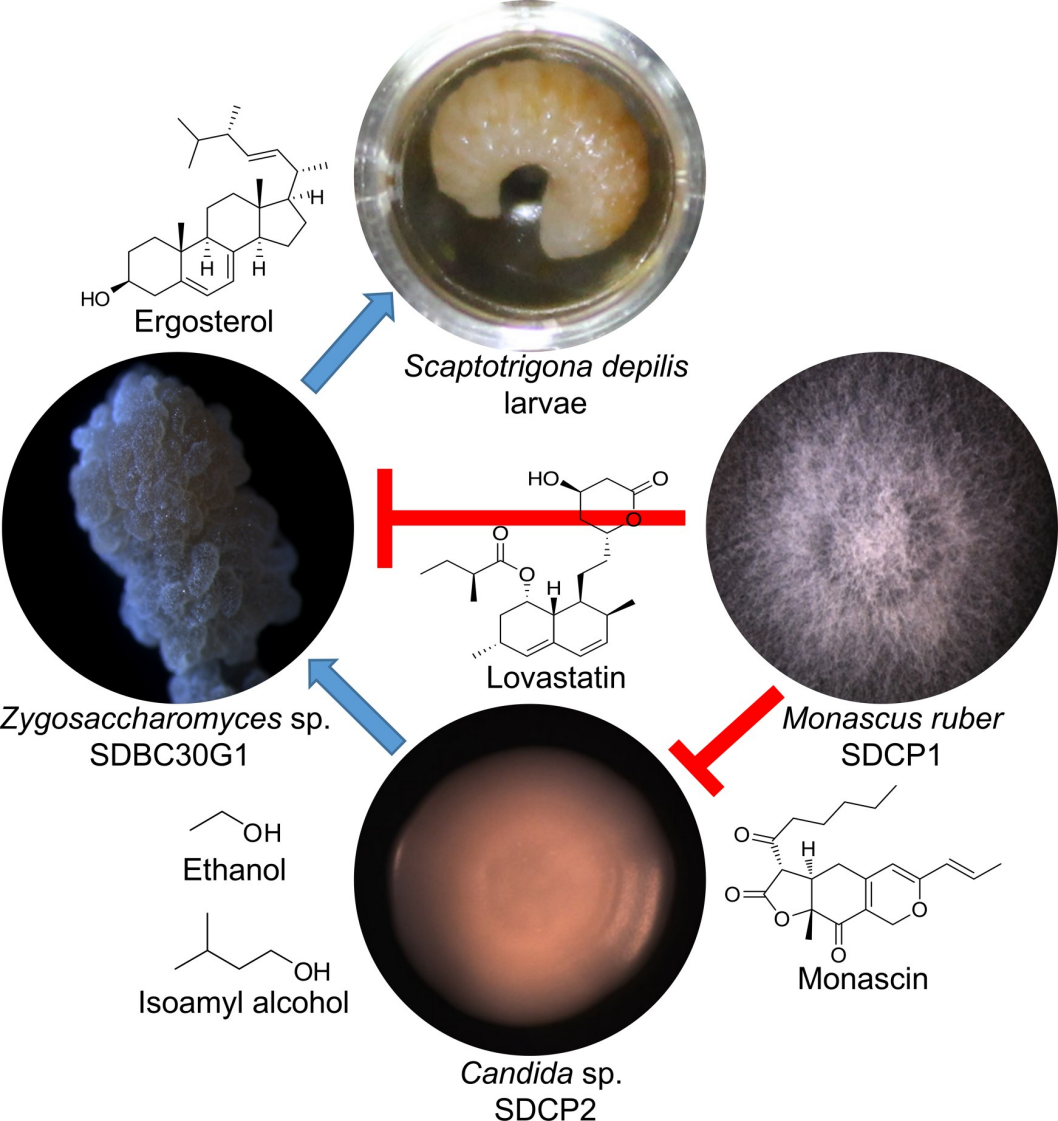
*Scaptotrigona depilis*-associated fungal community interactions. Depiction representing the stimulator that represents stimulatory (blue) and inhibitory (red) effects mediated by small molecules produced by the tripartite fungal community isolated from *Scaptotrigona depilis* brood cells.

The tripartite fungal community isolated from *S*. *depilis* brood cells interact using small molecules, interfering with the growth of each other, and these molecular interactions can impact bee survival. The stimulatory effects of *Candida* sp. SDCP2 VOCs on *Zygosaccharomyces* sp. SPBC30G1 growth seem to be an advantage to *S*. *depilis* larvae, since they depend on this fungus as a steroid source to develop [1]. Yet, *M*. *ruber* SDCP1, through the production monascin and lovastatin, can have an antagonistic effect on *Candida* sp. SDCP2 and *Zygosaccharomyces* sp. SDBC30G1, respectively (Fig 3).

The role that microorganisms play in insect development [1], protection [18,27–29] and communication [30–32] highlights the importance of studying these symbiotic microbial communities. In the case of social bees, such as honeybees and stingless bees, the knowledge about the associated microbiome can help the development of policies to control these pollinators’ disappearance caused by pesticides with antimicrobial activity. These microbes can suffer with toxic pesticides applied in agriculture, causing dangerous changes in the colony fitness and perturbing bee’s health [6,33–35]. The association of *S*. *depilis* with brood cells fungi is pivotal for colony homeostasis. The existence of *Candida* sp. and *M*. *ruber* in brood provisions seems to be as important as the presence of *Zygosaccharomyces* sp. for *S*. *depilis* development and life cycle.

## Materials and methods

### Microbial isolation

*Scaptotrigona depilis* colonies used for microbial isolation were maintained in wooden hives at University of São Paulo, Faculty of Philosophy, Sciences and Letters of Ribeirão Preto, Ribeirão Preto, São Paulo, Brazil (SISBIO authorization 46555–5, CNPq process 010936/2014-9). The collections were made from 2013 to 2016. Samples from brood cells cerumen were carefully collected and aseptically plated on Potato Dextrose Agar (PDA) containing streptomycin and penicillin (12.5 g/L). For *Zygosaccharomyces* sp. SDBC30G1 isolation, samples from the white microorganism growing inside *S*. *depilis* brood cells were plated aseptically on 30G agar medium (pH 6.0) [1]. The plates were incubated at 30°C until microbial growth was visible (3–7 days) to proceed with the purification. The stocks were kept at -80°C in solutions of PDB or 30G liquid medium plus 20% glycerol.

### DNA extraction and sequencing

DNA extractions from isolated strains were performed using FastDNA SPIN Kit for soil (MP), according the manufacturer instructions and quantified using NanoDrop. The isolated fungi were identified by 18S rRNA gene sequencing. The primers 18SF (5’-ATTGGAGGGCAAGTCTGGTG-3’) and 18SR (5’-CCGATCCCTAGTCGGCATAG-3’) were used for *Candida* sp. SDCP2. For *Monascus ruber* SDCP1 and *Zygosaccharomyces* sp. SDBC30G1, we used the primers NS1 (5’-GTAGTCATATGCTTGTCTC-3’) and NS4 (5’-CTTCCGTCAATTCCTTTAAG-3’). Data are available under accession numbers KX999554, KX999555 and KX999557 in the National Center for Biotechnology Information (NCBI).

### Co-cultures in agar

Co-cultures were performed in 15GF agar medium (pH 6.0 and pH 4.5) [1]. For this, 3 mL of the media were transferred to 6-well plates. *Candida* sp. SDCP2 was reactivated in PDB medium for 48 h at 120 rpm and 30°C. After reactivation, an inoculum of OD_600_ 0.06 was prepared. *Monascus ruber* SDCP1 was reactivated in PDA medium for seven days at 30°C. The spores of this fungus were collected and counted with the aid of a Neubauer chamber to prepare an inoculum containing 1x10^6^ spores/mL. *Zygosaccharomyces* sp. SDBC30G1 was reactivated in 30G agar medium (pH 6.0) for 10–15 days at 30°C. After this time, an inoculum of OD_600_ 0.06 was prepared. For all microorganisms, 2 μL of the inoculum were transferred to the culture media. The plates were incubated at 30°C for seven days. The experiments were made in duplicate.

### Medium acidification by *Candida* sp. SDCP2 and larval food pH

*Candida* sp. SDCP2 was cultured in duplicate in 100 mL of PDB (500 mL Erlenmeyer flask, without shaking, initial inoculum of OD_600_ 0.002) at 30°C for 3 days. The pH of the supernatant was measured using a direct reading pH stick. The pH of *S*. *depilis* larval food was measured using a pH meter (GEHAKA). The result was expressed as the average of the pH from four samples of larval food collected in different *S*. *depilis* colonies.

### Volatiles from *Candida* sp. SDCP2

A monoculture of *Candida* sp. SDCP2 was grown in 100 mL of 30G broth (pH 6.0) [1] (500 mL Erlenmeyer flask, without shaking, 30°C, initial inoculum of OD_600_ 0.002) for 10 days. Then, the culture was centrifuged for 10 min (6000 rpm at 4°C) and filtered sterilized to obtain the cell-free supernatant.

To test the stimulating capacity of the cell-free supernatant from *Candida* sp. SDCP2 on *Zygosaccharomyces* sp. SDBC30G1 growth, we spotted 10 and 20 μL of this supernatant on top of a fresh inoculated culture of *Zygosaccharomyce*s sp. SDBC30G1 in a Petri dish containing 25 mL of 30G agar medium, pH 6.0. *Zygosaccharomyce*s sp. SDBC30G1 inoculum was prepared from a 10–15 days-old culture in a Petri dish containing 30G agar medium, pH 6.0 (100 μL inoculum of OD_600_ 0.1), and plated using an inoculating loop. The control was made by replacing the cell-free supernatant from *Candida* sp. SDCP2 for 30G broth (pH 6.0). The Petri dishes were incubated at 30°C for 3–5 days.

For VOCs experiments, the supernatant (3 mL) from *Candida* sp. SDCP2 was poured inside small Petri dishes (10 x 35 mm). In other Petri dishes containing 3 mL of 30G agar medium (pH 6.0) each, 25 μL of *Zygosaccharomyce*s sp. SDBC30G1 inoculum (OD_600_ 0.02) were plated using an inoculating loop. This inoculum was prepared from a 24 h culture of *Zygosaccharomyce*s sp. SDBC30G1 in 2 mL of 30G broth (pH 6.0) at 200 rpm shaking and 30°C (culture started from 10–15 days-old culture in a Petri dish containing 30G agar medium, pH 6.0). The plates with *Zygosaccharomyce*s sp. SDBC30G1 were placed on top of plates containing *Candida* sp. SDCP2 supernatant, the plates were sealed together with parafilm and incubated individually inside plastic bags at 30°C [36]. Sterile 30G liquid medium (pH 6.0), instead of *Candida* sp. SDCP2 supernatant, was used as a control. After 3–5 days, the growth of *Zygosaccharomyces* in the presence of *Candida* sp. SDCP2 supernatant was compared to the control. This experiment was made in triplicate.

### Headspace analyses

Headspace analysis of *Candida* sp. SDCP2 supernatant was performed at Small Molecules Mass Spectrometry Facility (Faculty of Arts and Sciences Harvard University) using an Agilent 6890 gas chromatograph and CTC CombiPAL autosampler. A DB-624UI column (30m x 0.32mm x 1.80um) was used, at flow rate of 2.3 mL/min, injection temperature of 220°C, interface at 220°C, ion source of 200°C, and *m/z* 10–500. Initial temperature was set at 30°C for 6 min, then increased at 10°C/min to 150°C, and then at 50°C/min to 250°C, and kept at 250°C for 15 min. The split ratio was 200 and headspace method adopted was 80°C for 10 min. The reads were compared to the NIST MS search 2.0 library.

*Scaptotrigona depilis* brood cells headspace analysis was performed at Research Support Center in Natural and Synthetic Products (NPPNS), University of São Paulo, using a Shimadzu GC-2010 Plus gas chromatograph and the auto sampler AOC 5000 with 2.5 mL syringe. A ZB-624 column (30m x 0.32mm x 1.80um) was used, at flow rate of 2.68 mL/min, injection temperature of 260°C, interface at 220°C, ion source of 200°C, and *m/z* 10–200. Initial temperature was set at 30°C for 6 min, then increased at 10°C/min to 150°C, and then at 50°C/min to 250°C, and kept at 250°C for 15 min. The split ratio was 5 and headspace method adopted was 80°C for 45 min. The reads were compared to the NIST 11 library.

### Co-cultures of *Monascus ruber* SDCP1 and *Candida* sp. SDCP2 in liquid medium

*Monascus ruber* SDCP1 and *Candida* sp. SDCP2, isolated from cerumen of *S*. *depilis* brood cells, were grown in PDA at 30°C for 7 days. After this period, two fungal-agar fragments (diameter of approximately 6 mm) were transferred to 50 mL Falcon tubes containing 20 mL of Potato Dextrose Broth (PDB). The cultures were incubated at 30°C with 120 rpm shaking and, after 48 h, were combined in Erlenmeyer flasks (500 mL) containing 150 mL of PDB. Monocultures of each microorganism were performed as controls. To analyze the production of metabolites, 10 mL of each culture were collected daily, centrifuged and filtered, and a sample of 1 mL was extracted with solid phase extraction (SPE) cartridges (C_18_, 50 mg, Waters) using 1 mL of methanol. The organic extracts were filtered and 4 μL were analyzed by UPLC 1290 Infinity (Agilent Technologies) coupled to 6530 ESI-Q-TOF (Agilent Technologies) operating in the positive mode. A 10 x 4.6 mm column, Luna 5 mm C_18_ (Phenomenex) was used, with acetonitrile:water gradient as mobile phase, flow rate of 0.7 mL/min (starting with 10% of acetonitrile, maintaining this percentage for 1 min, then reaching 100% of acetonitrile in 18 min, and maintaining this percentage for 5 min).

### Determination of minimum inhibitory concentration (MIC) and minimum fungicidal concentration (MFC) of monascinol and monascin produced by *M*. *ruber* SDCP1

The experiments were carried out according to the conditions recommended by CLSI (Formerly NCCLS) [37]. *Candida* sp. SDCP2 cultures were incubated in Petri dishes containing PDA for 48 h at 30°C. Cultures of *C*. *albicans* ATCC MYA—2876 were reactivated in Petri dishes containing LB, Miller agar [38] for 24 h at 37°C. The inoculums were prepared as follows, using PDB for *Candida* sp. SDCP2 and LB-Miller for *C*. *albicans* ATCC MYA—2876. When OD_600_ of the turbid solution was 0.08, 67 μL were transferred to 933 μL of culture medium. This final volume (1.0 mL) was transferred to another tube containing 9.0 mL of culture medium and vortexed. Then, 2.0 mL were transferred to a tube containing 10 mL of culture medium, and these inoculums were used in the treatments.

Cycloheximide (Sigma-Aldrich) was used as a positive control for inhibiting *Candida* sp. SDCP2, and clotrimazole (Sigma-Aldrich) was used for *C*. *albicans* ATCC MYA—2876. To prepare the positive controls solutions, 3.2 mg of each antifungal were dissolved in 0.5 mL of dimethyl sulfoxide (DMSO). Then, 16 μL were transferred to 144 μL of PDB or LB-Miller media and vortexed. Finally, 80 μL were transferred to the first well and serially diluted.

Monascin and monascinol used in these assays were obtained from *M*. *ruber* SDCP1 culture (90 g of rice, 120 mL of water in 500 mL Erlenmeyer flask, incubated for 30 days at 30°C, followed by ethyl acetate extraction and HPLC isolation) and characterized by NMR and ESI-HRMS (S7 and S8 Figs, S1 Data). Spectral data are in agreement with previously published data for monascin [39] and monascinol [40]. Each metabolite (0.5 mg) was dissolved in 62.5 μL of DMSO (8000 μg/mL). Then, 10 μL of these solutions were mixed with 70 μL of culture medium. The final solutions (80 μL) were transferred to the first well and serial dilution was performed. Then, 20 μL of the inoculum was added to each well, achieving the final concentration of 400 μg/mL in the first well. The plate containing *C*. *albicans* ATCC MYA—2876 was maintained for 24 h at 37°C, and the plate with *Candida* sp. SDCP2 was incubated for 48 h at 30°C. After the incubation time, the OD_600_ of the cultures were checked to determine microbial growth. The experiments were performed in duplicate.

To determine the minimal fungicide concentration (MFC), a sample of each well was removed with sterile toothpicks and transferred to Petri dishes containing PDA or LB agar culture media. The plates were incubated for 48 h at 30°C or 24 h at 37°C for *Candida* sp. SDCP2 and *C*. *albicans* ATCC MYA—2876, respectively, and were visually inspected for the presence or absence of growth.

### Lovastatin effects on *Zygosaccharomyces* sp. SDBC30G1 growth

*M*. *ruber* SDCP1 was cultured in 30G agar plates for seven days at 30°C. After this period, the culture was extracted with ethyl acetate, the solvent was dried using rotary evaporator, yielding a crude extract. The HRESIMS analysis of the extract was carried out using a Bruker Maxis Impact HD LC-q-TOF Mass Spectrometer equipped with an uHPLC system showing lovastatin as the major compound (Lovastatin *m/z* 405.2640 [M+H]^+^, calculated mass for C_24_H_36_O_5_ 405.2636 [M+H]^+^, error 1.1 ppm). The production of lovastatin by *M*. *ruber* SDCP1 was further confirmed by retention time comparison with lovastatin standard (Sigma-Aldrich) using a Quadrupole 6130 (Agilent Technologies) equipped with HPLC-DAD-MS 1200 series (Agilent Technologies).

Lovastatin standard (Sigma-Aldrich) was used to determine its effects on *Zygosaccharomyces* sp. SDBC30G1 growth. Stock solutions were prepared in ethanol (12500 μM, 6250 μM and 3125 μM) and 5 μL of each solution were transferred to the corresponding wells in a 6-well plate containing 4995 μL of freshly inoculated *Zygosaccharomyces* sp. SDBC30G1 cultures (OD_600_ 0.1, prepared from a 10–15 days-old culture of *Zygosaccharomyces* sp. SDBC30G1 in 30G agar medium at 30°C) in 15GF pH 4.5 medium [1]. The final concentrations of lovastatin tested were 12.5 μM, 6.25 μM and 3.125 μM. For the control, we transferred 5 μL of pure ethanol to the correspondent wells. The plates were incubated at 30°C for seven days.

## Supporting information

S1 Fig *Scaptotrigona depilis* brood cell. (PDF)

S2 Fig Interactions between *Zygosaccharomyces* sp. SDBC30G1 and *Candida* sp. SDCP2. (PDF)

S3 Fig GC-MS analysis of VOCs from *Candida* sp. SDCP2 supernatant. (PDF)

S4 Fig GC-MS analysis of VOCs from *Scaptotrigona depilis* brood cells. (PDF)

S5 Fig Interactions between the fungi isolated from *Scaptotrigona depilis* brood cells. (PDF)

S6 Fig LC-MS (positive mode) analysis of *Monascus ruber* SDCP1 monoculture in 30G (pH 6.0) medium extracted with ethyl acetate after seven days at 30°C. (PDF)

S7 Fig ^1^H NMR spectrum of monascinol (CDCl_3_, 500 MHz) produced by *Monascus ruber* SDCP1. (PDF)

S8 Fig ^1^H NMR spectrum of monascin (CDCl_3_, 500 MHz) produced by *Monascus ruber* SDCP1. (PDF)

S1 Video Time lapse of a co-culture between *Monascus ruber* SDCP1 and *Candida* sp. SDCP2 showing the pigmentation stimulus caused by the yeast. (MP4)

S1 Data Raw NMR data of monascin and monascinol (CDCl_3_, 500 MHz). (ZIP)
