# Supplementary material for: Microbial community modulates growth of symbiotic fungus required for stingless bee metamorphosis
Source: PLoS One. 2019 Jul 25;14(7):e0219696. doi: 10.1371/journal.pone.0219696 (PMC6657851; doi:10.1371/journal.pone.0219696)

S1 Fig.

Opened brood cell showing *Zygosaccharomyces* sp. growth (white pseudohyphae), cerumen in the board of the brood cell, larval food and the larva.

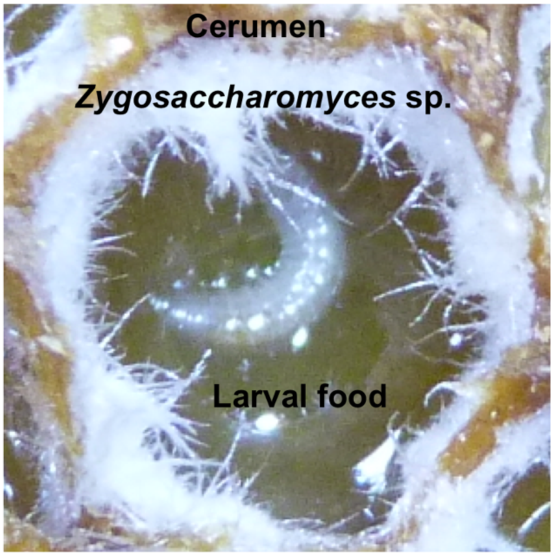

Supplement: S1 Fig — (PDF) [file pone.0219696.s001.pdf]
