# Supplementary material for: Microbial community modulates growth of symbiotic fungus required for stingless bee metamorphosis
Source: PLoS One. 2019 Jul 25;14(7):e0219696. doi: 10.1371/journal.pone.0219696 (PMC6657851; doi:10.1371/journal.pone.0219696)

S2 Fig.

A. *Zygosaccharomyces* sp. SDBC30G1 monoculture (left), *Candida* sp. SDPC2 and *Zygosaccharomyces* sp. SDBC30G1 co-culture (middle), *Candida* sp. SDPC2 monoculture (rigth), after 7 days cultured in 15GF medium (pH 4.5).

B. *Zygosaccharomyces* sp. SDBC30G1 monoculture (left), *Candida* sp. SDPC2 and *Zygosaccharomyces* sp. SDBC30G1 co-culture (middle), *Candida* sp. SDPC2 monoculture (rigth), after 7 days cultured in 15GF medium (pH 6.0).

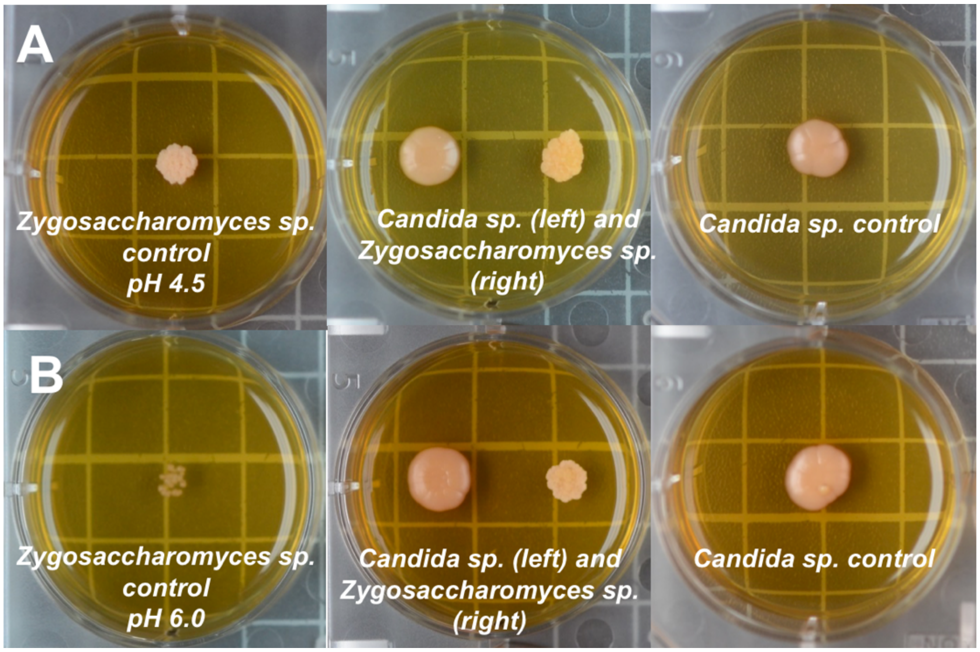

C. Culture of *Zygosaccharomyces* sp. SDBC30G1 with 20 μL and 10 μL *Candida* sp. SDCP2 supernatant (right). The stimulus observed in comparison with the control (left) was spread, indicating the presence of stimulatory VOCs.

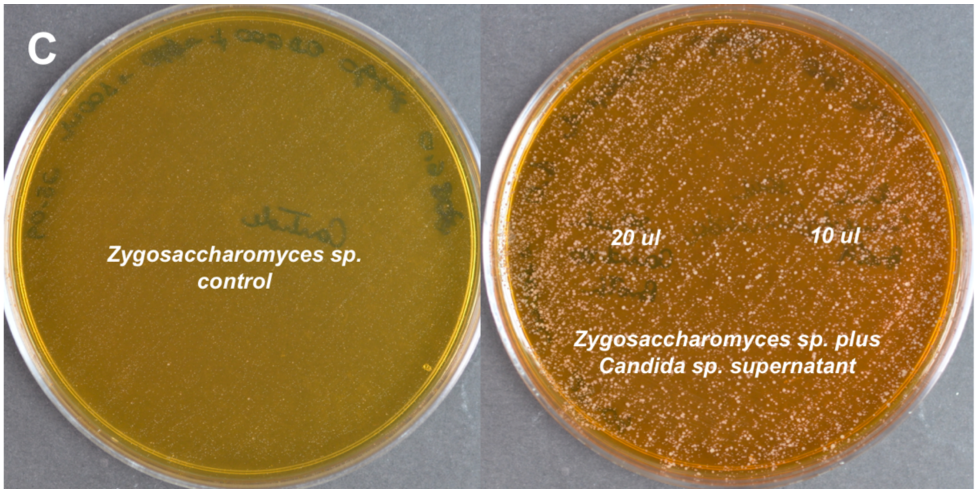

D. Culture of *Zygosaccharomyces* sp. SDBC30G1 control (left), culture of *Zygosaccharomyces* sp. SDBC30G1 after contact with VOCs produced by *Candida* sp. SDCP2 (middle). *Zygosaccharomyces* sp. SDBC30G1 cells presenting flotation phenotype in the test (right).

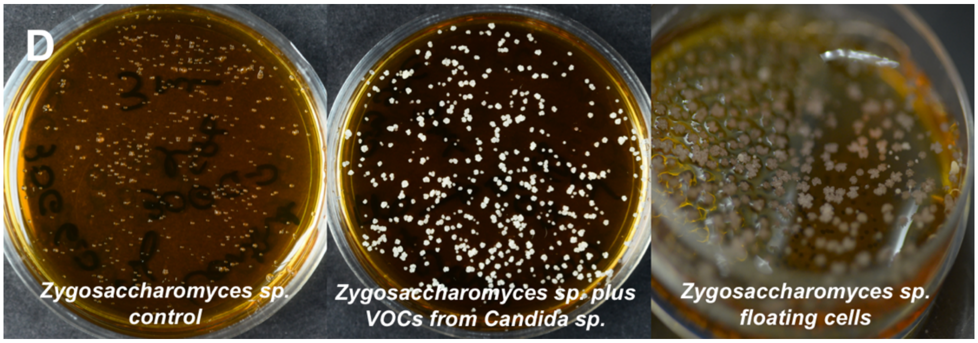

Supplement: S2 Fig — (PDF) [file pone.0219696.s002.pdf]
