# Supplementary material for: Microbial community modulates growth of symbiotic fungus required for stingless bee metamorphosis
Source: PLoS One. 2019 Jul 25;14(7):e0219696. doi: 10.1371/journal.pone.0219696 (PMC6657851; doi:10.1371/journal.pone.0219696)

S3 Fig.

A. Headspace analyses of *Candida* sp. SDCP2 supernatant showing ethanol (C1) as the most intense peak (2.1 min), followed by isoamyl alcohol (C2) (10.9 min).

B. Analysis of 30G liquid medium as control.

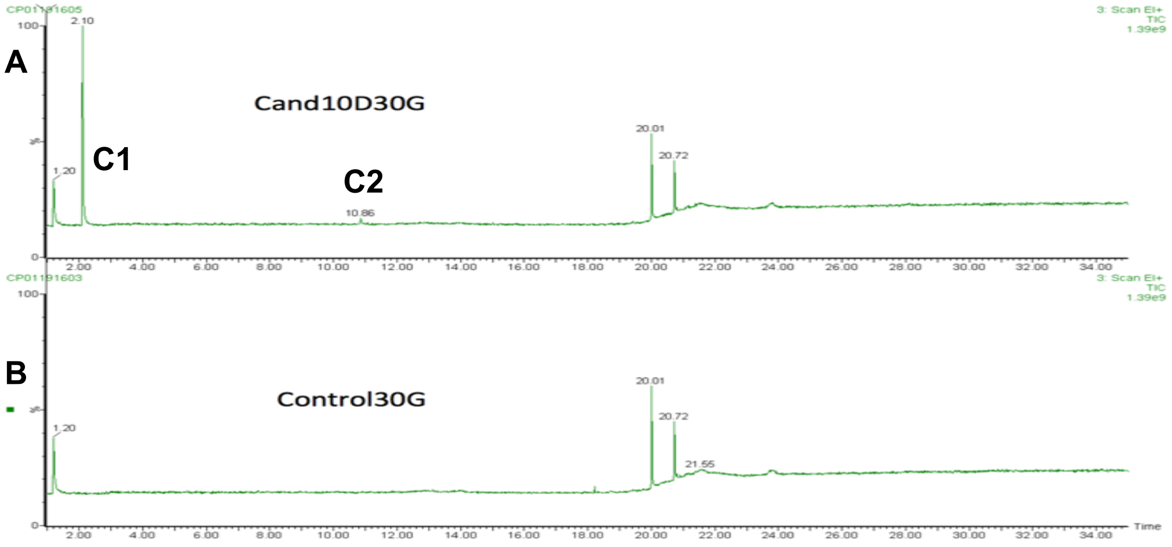

C. Characterization of ethanol (C1), present in *Candida* sp. SDCP2 supernatant, using NIST library.

D. Characterization of isoamyl alcohol (C2), present in *Candida* sp. SDCP2 supernatant, using NIST library.

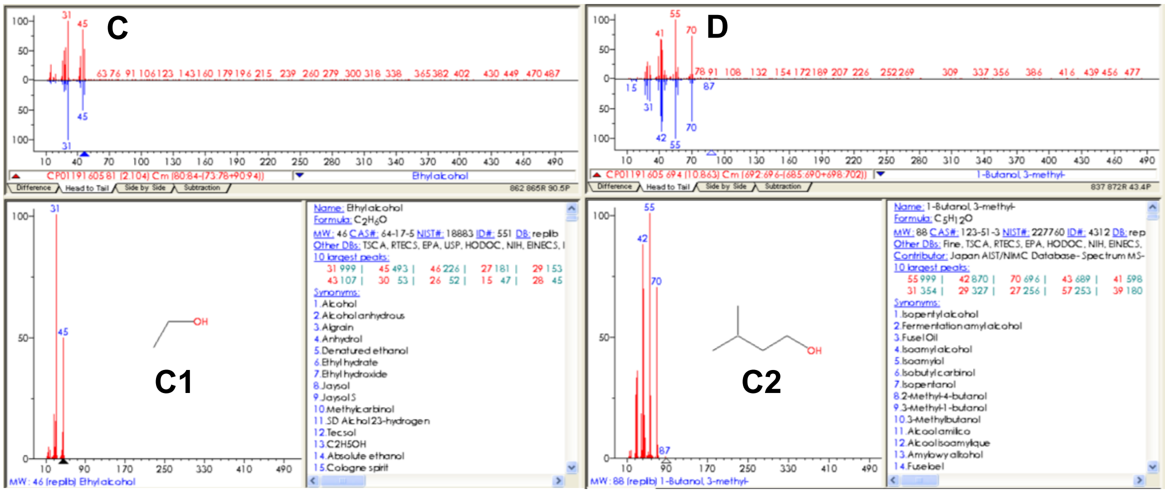

Supplement: S3 Fig — (PDF) [file pone.0219696.s003.pdf]
