# Supplementary material for: Microbial community modulates growth of symbiotic fungus required for stingless bee metamorphosis
Source: PLoS One. 2019 Jul 25;14(7):e0219696. doi: 10.1371/journal.pone.0219696 (PMC6657851; doi:10.1371/journal.pone.0219696)
Supplement: S4 Fig — (PDF) [file pone.0219696.s004.pdf]

S4 Fig.

A. Headspace analysis of *S. depilis* brood cells VOCs, showing ethanol (C1) is the highest signal (2.2 min). Isoamyl alcohol (C2) is also detected (11.2 min). Presence of ethyl acetate (6.0 min) and acetic acid (9.0 min).

B. Headspace analysis of the vial as control.

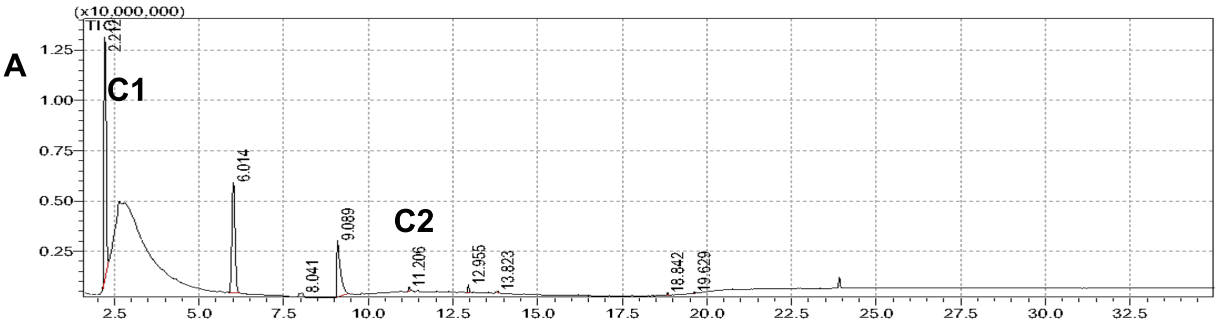

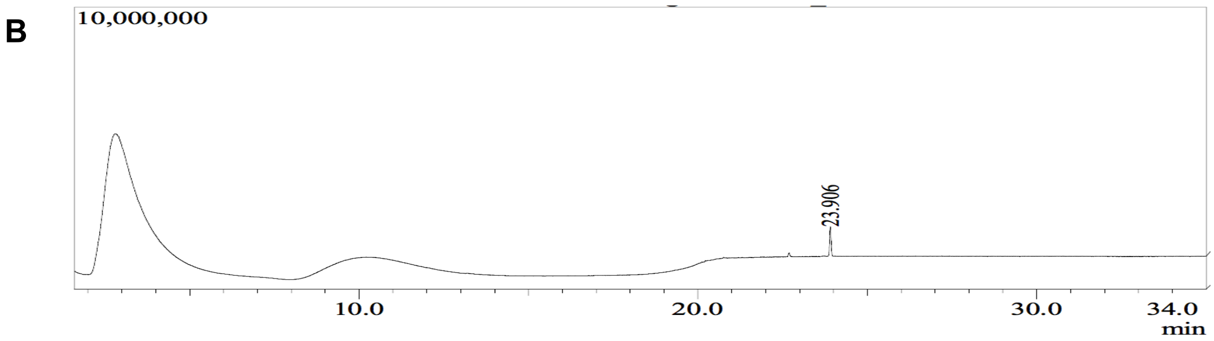

C. Characterization of ethanol (C1), present in *S. depilis* brood cells, using NIST library.

D. Characterization of isoamyl alcohol (C2), present in *S. depilis* brood cells, using NIST library.

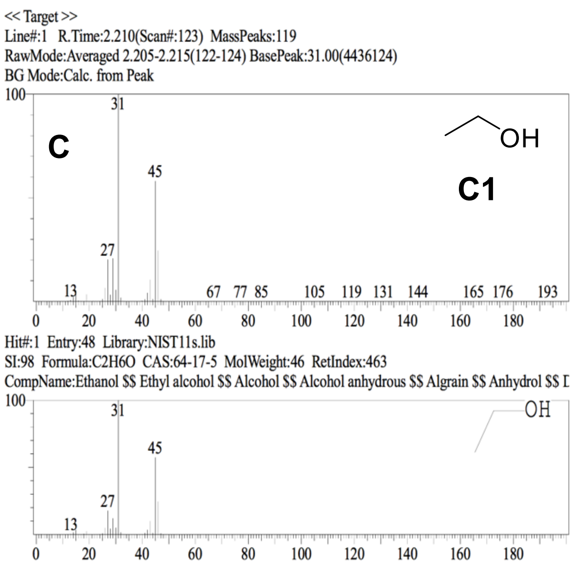

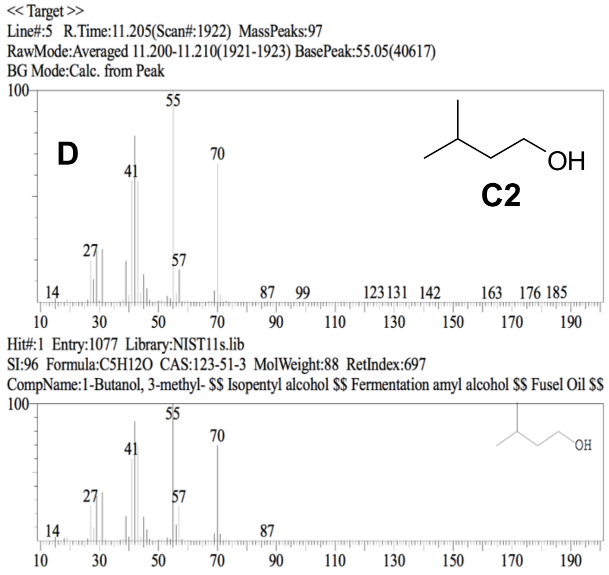

The six most abundant compounds identified:

Peak Report TIC

Peak#	R.Time	Area	Area%	Name
1	2.212	57731868	49.44	Ethanol
2	6.014	35270049	30.21	Ethyl Acetate
3	8.041	1740630	1.49	Butanal, 3-methyl- (CAS) 3-Methylbutar
4	9.089	19911097	17.05	Acetic acid
5	11.206	613918	0.53	1-Butanol, 3-methyl-
6	12.955	999479	0.86	Propanoic acid, 2-hydroxy-, ethyl ester
