# Supplementary material for: Microbial community modulates growth of symbiotic fungus required for stingless bee metamorphosis
Source: PLoS One. 2019 Jul 25;14(7):e0219696. doi: 10.1371/journal.pone.0219696 (PMC6657851; doi:10.1371/journal.pone.0219696)

S5 Fig.

Monocultures and co-cultures of *M. ruber* SDCP1 (M); *Candida* sp. SDCP2 (C) and *Zygosaccharomyces* sp. SDBC30G1 (Z)

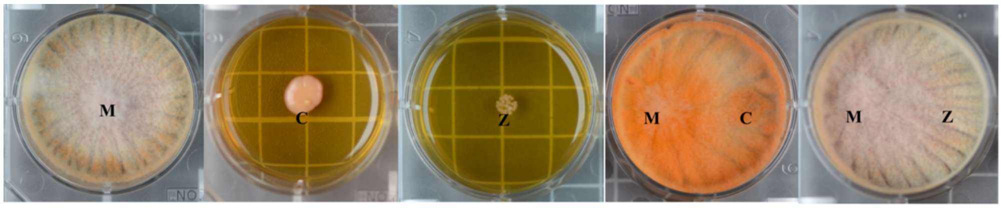

A. Extracted-ion chromatogram (LC-HRMS, positive mode) of the co-culture between *M. ruber* SDCP1 and *Candida* sp. SDCP2 in liquid medium, searching for monascin *m/z* 359.1853 (retention time 10.6 min)

B. Extracted-ion chromatogram (LC-HRMS, positive mode) of the co-culture between *M. ruber* SDCP1 and *Candida* sp. SDCP2 in liquid medium, searching for monascinol *m/z* 361.2010 (retention time 9.6 min).

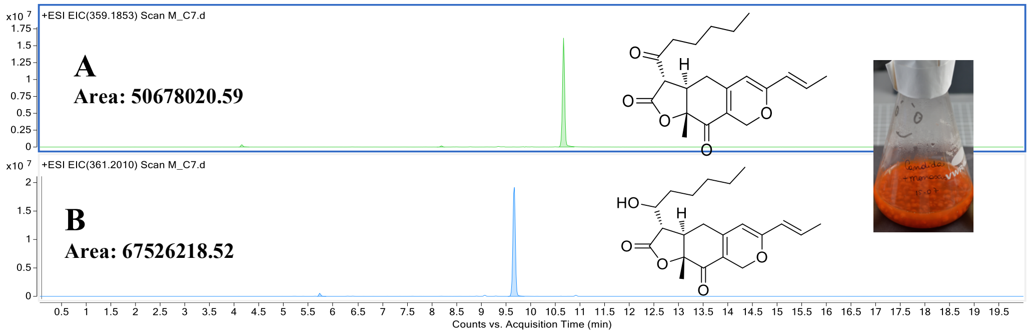

C. Extracted-ion chromatogram (LC-HRMS, positive mode) of *M. ruber* SDCP1 monoculture in liquid medium, searching for monascin *m/z* 359.1853 (retention time 10.6 min). Obs: unspecific pics were disregarded by retention time comparison.

D. Extracted-ion chromatogram (LC-HRMS, positive mode) of *M. ruber* SDCP1 monoculture in liquid medium, searching for monascinol *m/z* 361.2010 (retention time 9.6 min).

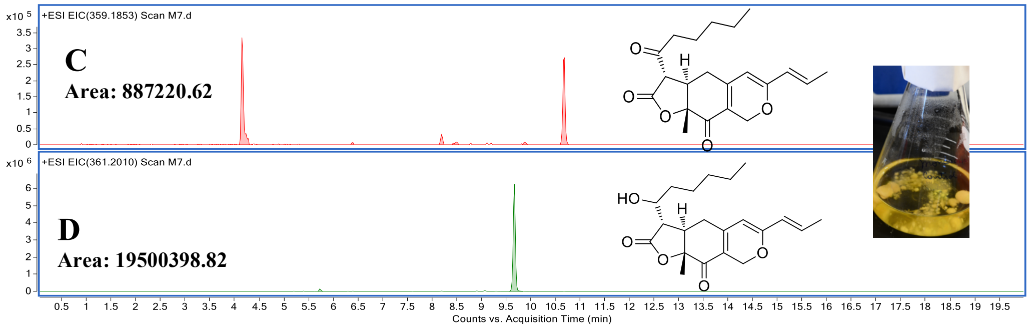

Supplement: S5 Fig — (PDF) [file pone.0219696.s005.pdf]
