# Supplementary material for: Microbial community modulates growth of symbiotic fungus required for stingless bee metamorphosis
Source: PLoS One. 2019 Jul 25;14(7):e0219696. doi: 10.1371/journal.pone.0219696 (PMC6657851; doi:10.1371/journal.pone.0219696)

S6 Fig.

A. The major peak corresponds to lovastatin (11.5 - 11.6 min).

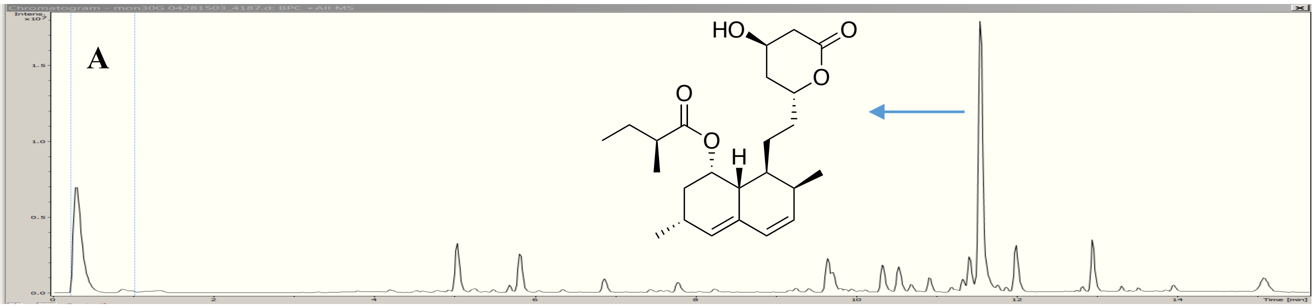

B. Extracted-ion chromatogram, searching for *m/z* of lovastatin (*m/z* 405 $[M+H]^+$).

C. Lovastatin HRMS *m/z* 405.2640 $[M+H]^+$, calculated mass for $C_{24}H_{36}O_5$ 405.2636 $[M+H]^+$, error 1.1 ppm. It is possible to visualize the adducts with ammonium (*m/z* 422) and sodium (*m/z* 427).

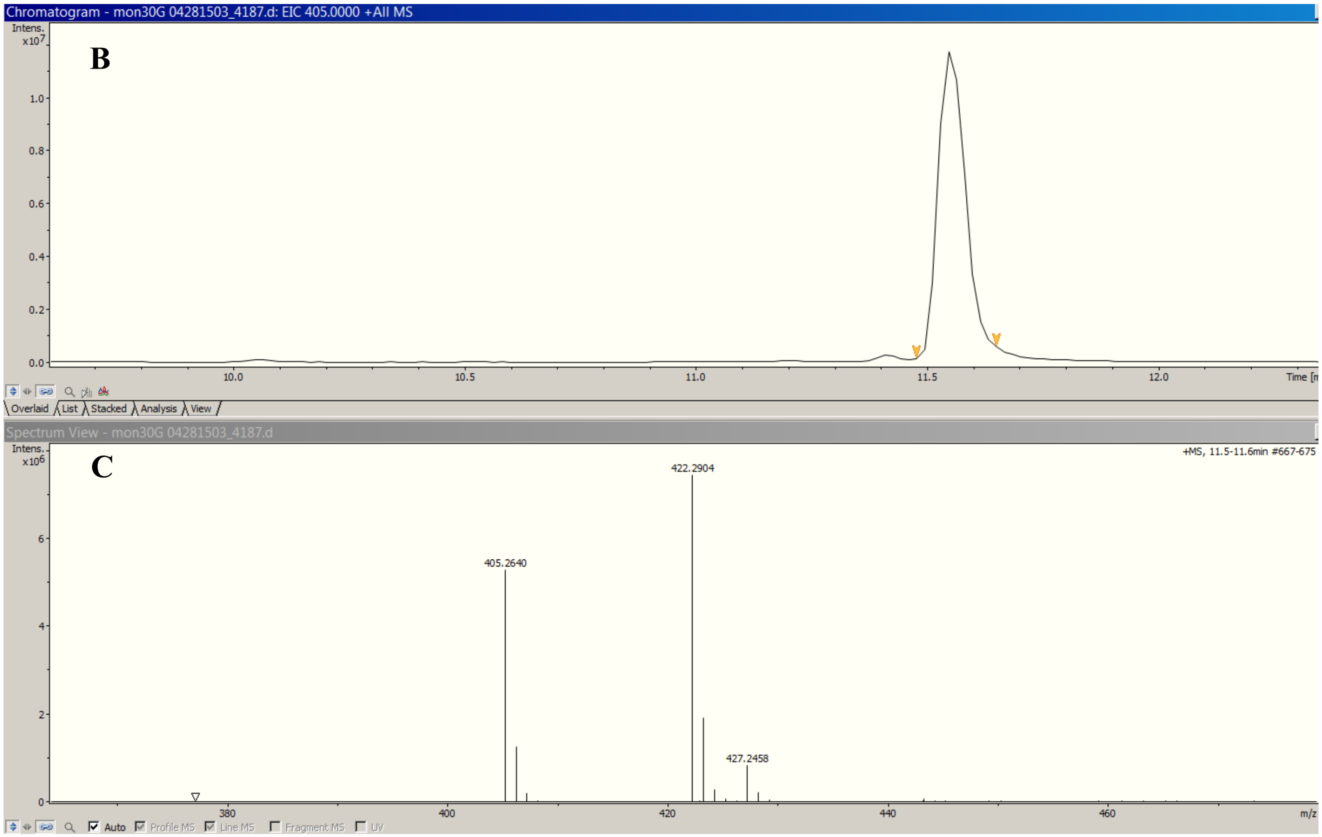

Supplement: S6 Fig — (PDF) [file pone.0219696.s006.pdf]
