# Supplementary figures and images for: Microbial community modulates growth of symbiotic fungus required for stingless bee metamorphosis
Source: PLoS One. 2019 Jul 25;14(7):e0219696. doi: 10.1371/journal.pone.0219696 (PMC6657851; doi:10.1371/journal.pone.0219696)

S7 Fig.

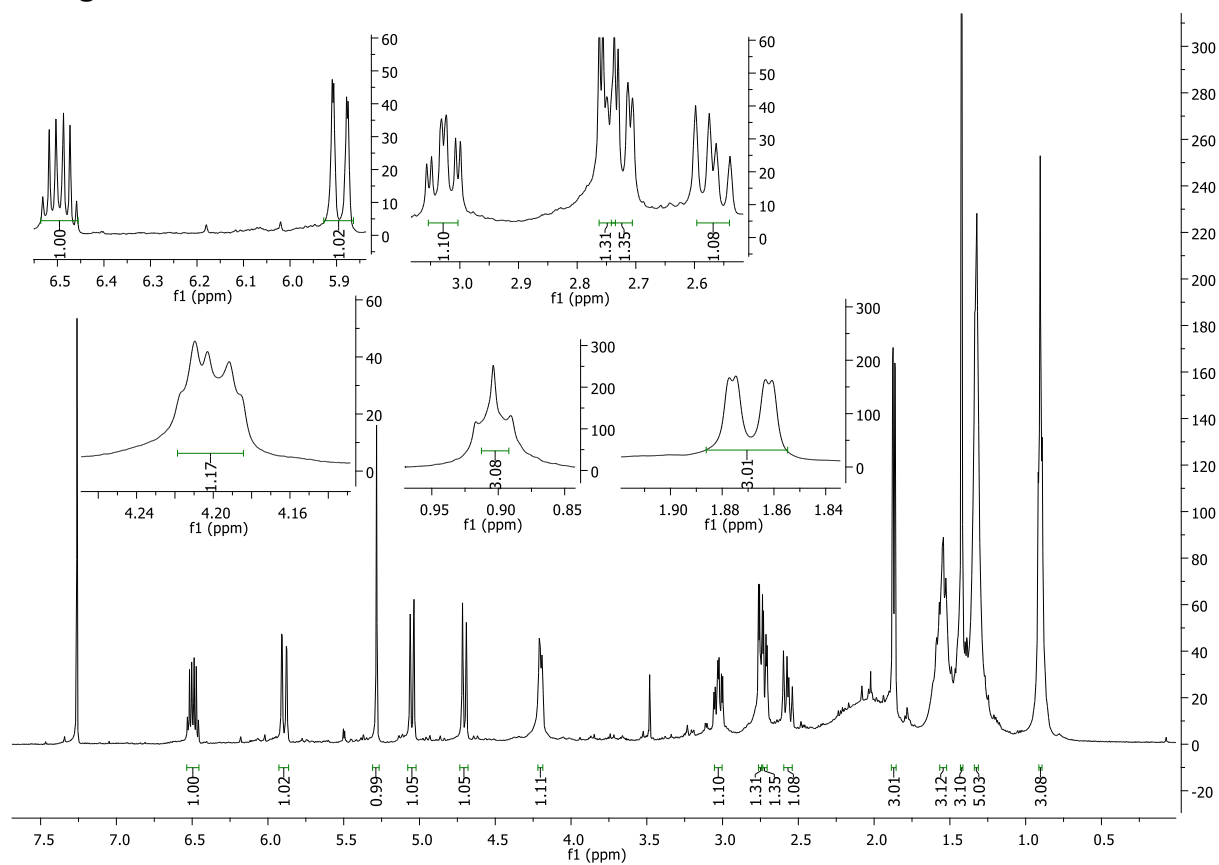

Supplement: S7 Fig — (PDF) [file pone.0219696.s007.pdf]

S8 Fig.

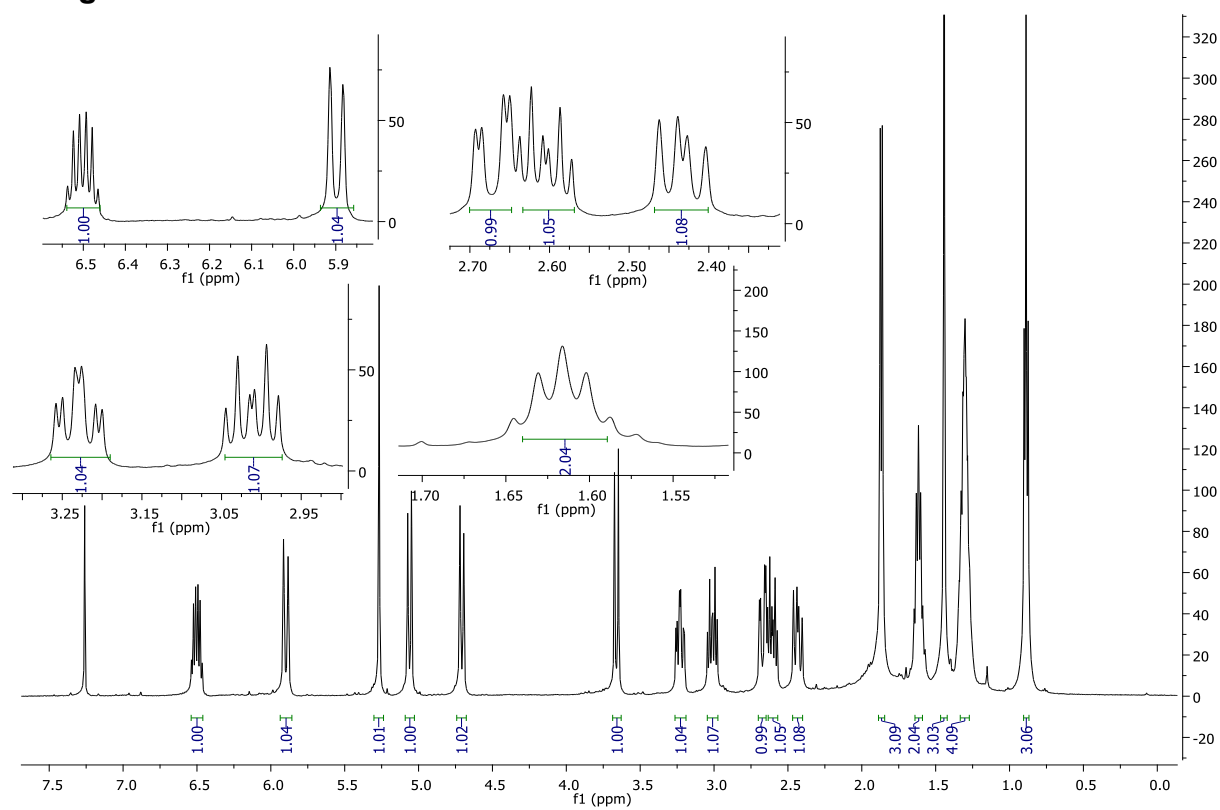

Supplement: S8 Fig — (PDF) [file pone.0219696.s008.pdf]
